# Encapsulation of Cumin (*Cuminum cyminum* L.) Seed Essential Oil in the Chickpea Protein–Maltodextrin Matrix

**DOI:** 10.1021/acsomega.2c07184

**Published:** 2023-01-18

**Authors:** Onur Atli, Asli Can Karaca, Beraat Ozcelik

**Affiliations:** Department of Food Engineering, Faculty of Chemical and Metallurgical Engineering, Istanbul Technical University, 34469Istanbul, Turkey

## Abstract

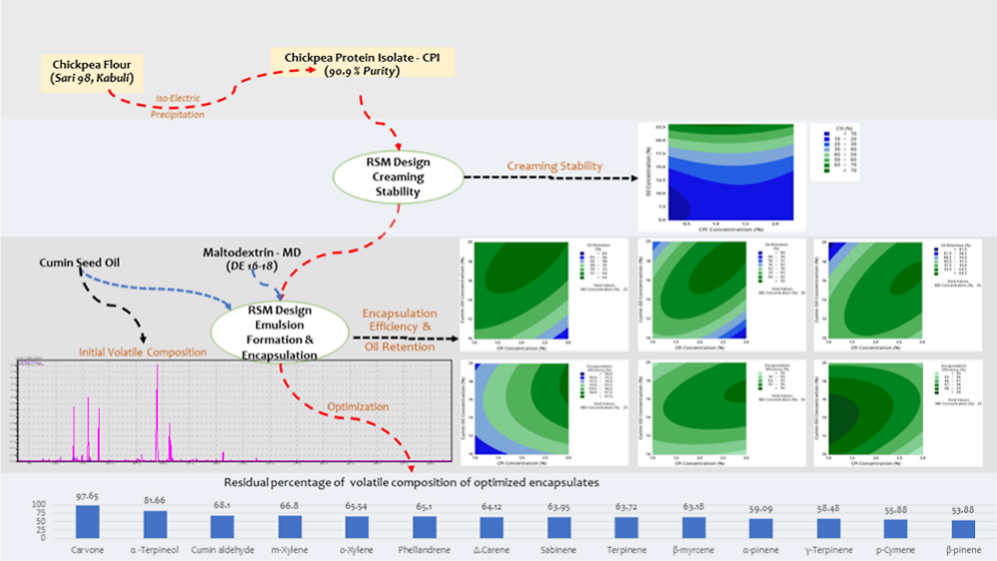

Isoelectrically precipitated chickpea protein isolate (CPI) and its combination with maltodextrin (MD) were investigated for the ability to form and stabilize cumin seed oil emulsions. Solubility, net surface charge, emulsion activity/stability indices, and creaming stability of CPI at a pH of 3.0–9.0 were evaluated. Optimum conditions for minimum cream separation were identified as: 0.19% CPI and 6.83% oil concentrations. Cumin (*Cuminum cyminum* L.) seed essential oil was microencapsulated within the CPI–MD matrix via spray drying. Effects of CPI–MD matrix formulation on the physicochemical characteristics and volatile composition of the microencapsules were investigated. CPI–MD matrices had positive effects on microcapsule properties such as relatively lower surface oil, higher encapsulation efficiency (EE), and oil retention. Approximately 86.6–96.4% oil retention and 90.9–98.4% EE were achieved. Optimum conditions for maximized oil retention (92.9%) and EE (98.6%) were identified as: 2.1% CPI, 14.8% essential oil, and 35% MD. GC–MS analysis of microcapsules was carried out to determine the changes in volatile composition during spray drying. Cymene, α-pinene, β-pinene, sabinene, terpinene, terpineol, phellandrene, and cumin aldehyde were determined as the major components. Optimized design showed the highest EE and minimal changes in the volatile composition of cumin seed essential oil.

## 1 Introduction

Microencapsulation provides improved delivery and stability for bioactive ingredients, compared to their liquid form because the degradation is inhibited by the coating medium. Polyunsaturated fatty acids and essential oils can be easily degraded by oxygen or heat and are required to be protected from environmental factors.^1^ The wall material is among the main factors affecting the functional properties of both the encapsulated product and the encapsulation process.^2^ The suitable choice of wall material majorly depends on physicochemical properties such as solubility, molecular weight, crystallinity, diffusibility, glass transition, film-forming capacity, and emulsifying properties.^3^ Most commonly used wall materials are maltodextrins (MDs), hydrophobically modified starch, and their combinations. In addition to carbohydrates, lipids (e.g., stearic acid and mono- and diglycerides) and proteins (e.g., whey proteins, pulse proteins, sodium caseinate, albumins, gelatin, and casein) are also used. However, their usage as wall materials has limited availability due to their relatively lower water solubility. On the other hand, addition of these substances on the complex matrix in a small amount has been shown to provide some benefits on the stability of encapsulated ingredients.^4^ MDs are considered as the most commonly applied wall materials, due to their optimum water solubility, low viscosity at higher concentrations, lower sugar content, adequate protection against oxidation, relatively lower cost, and neutral aroma.^5^ However, their poor emulsification properties limit their application in encapsulation of hydrophobic substances. Hence, to obtain more stable emulsions before the encapsulation, it is required to create complex matrices by using other substances. Therefore, to develop microcapsules with better protective walls, polysaccharides are generally combined with protein fractions to obtain complex matrices because their interaction provides better emulsification properties.^6^ Due to their amphiphilic nature, being both hydrophilic and hydrophobic, proteins are capable of unfolding at oil–water interactions, which leads them to form a viscoelastic coating layer around the dispersed oil droplets by lowering the interfacial tension.^7^ When they form a coating layer around the lipid globules, due to the partial denaturation, some hydrophilic proteins, that have an interaction with lipid globules, shift into the surface of the layer to interact with water. Due to their both emulsifying and film-forming properties, pulse proteins are considered as potential microencapsulation agents.^8^ Chickpea protein comprises globulins (56.0%), glutelins (18.1%), albumins (12.0%), prolamin (2.8%), and residual proteins.^9^ Additionally, the major globulin fraction present in chickpea is the legumin-like globulin (11S) and contains a lesser extent of vicilin (7S).^10^

Essential oils are considerably complex, volatile compounds naturally derived from organic sources such as aromatic plants. Essential oils are synthesized as secondary metabolites from aromatic plants such as thyme, peppermint, cinnamon, tea tree, rosemary, and cumin and are distinguished by their characteristic odor.^11^ However, essential oils are highly unstable against environmental conditions and can undergo oxidative deterioration and lose volatile components. To overcome the degradation of essential oils to sustain their biological and functional characteristics, microencapsulation is used as an effective strategy. Spray drying is among the most frequently applied microencapsulation methods in the food industry, due to its higher yield. Furthermore, compared to other encapsulation methods it is also relatively easy to apply, affordable, and easy to scale up with repeatability.^12^ The challenging factor in this process technology is the preserving the physical characteristics during drying. Encapsulation efficiency (EE) can be improved in spray-drying applications by using a proper core to wall ratio, which provides protection and mechanical stability by the wall material(s).

Cumin (*Cuminum cyminum* L.), an herbaceous plant from Apiaceae family, is among the most common spices worldwide, and it has originated from Eastern Mediterranean countries.^13^ Cumin seed essential oil is also known to possess several pharmacological properties, such as antimicrobial, antidiabetic, antiepileptic, anti-infertility, anticancerous, and immunomodulative effects due to the presence of active chemical constituents.^14^ Cumin commonly contains around 2–5% essential oil which has light brownish yellow color with a slightly bitter, aromatic flavor.^15^ The essential oil contains variable amount of cumin aldehyde 20–40%, and the odor is mainly attributed to cumin aldehyde, the major compound present in oil, α-pinene, β-pinene, cymene, terpinene, cumin aldehyde, and other related aldehydes.^14^ There are current studies on encapsulation of cumin seed essential oil using a variety of wall material matrix combinations including chitosan-caffeic acid,^16^ chitosan nanoparticles,^17,18^ cocoa butter, and cocoa butter substitutes.^19^ However, to the best of our knowledge, encapsulation of cumin seed essential oil in a plant-based wall material matrix via spray drying has not been reported up to date. The main goal of our study was to encapsulate cumin seed oil within a chickpea protein–MD matrix that would protect the volatile composition of the essential oil. Protein isolated from Sari 98, an Anatolia originated chickpea cultivar, was evaluated as a wall material for encapsulation of essential oils. The wall material combinations were characterized and feed emulsion formulations were optimized in order to yield the most stable feed emulsions that would result in increased EE and improved retention of volatile composition of cumin seed essential oil.

## 2 Materials and Methods

### 2.1 Materials

Chickpea (Sari 98, Kabuli) was supplied from a local store in a finely ground form. Cumin seed essential oil was kindly donated by Altes Ltd. (Antalya, Turkey), an essential oil manufacturing company, and MD (DE 12–16) was kindly donated by Cargill Inc. (Istanbul, Turkey). All chemicals used for the analysis were of reagent grade.

### 2.2 Protein Isolation

Protein isolates were prepared by using a modified version of the method of Karaca et al.^20^ Defatting of the sample was performed by using chickpea flour/hexane (1:1 w/v) in a magnetic stirrer for 30 min. The solution was then filtered through a 110 mm filter paper (Whatman International Ltd., Maidstone, UK). The defatting steps were repeated four times until the defatted flour was obtained. For removal of the excess amount of hexane in the mixture, defatted sample was air-dried in a fume hood. Afterward, defatted chickpea flour was dissolved in deionized water (1:10 w/v), pH was adjusted to 9.0 with 1.0 N NaOH, and stirred at 1000 rpm for 60 min at room temperature (20–23 °C). In order to collect the supernatant, the suspension was then centrifuged at 12,000*g* for 10 min at 4 °C using a Rotina 380R centrifuge (Andreas Hettich GmbH & Co. KG, Tuttlingen, Germany). Supernatants were collected and pH was adjusted to 4.5 with 1.0 N HCl, near the isoelectric point of chickpea protein, to precipitate the protein fraction in the solution. The protein was then recovered by centrifugation, collected, and stored at −55 °C for 20 h to obtain low moisture content chickpea protein isolate (CPI). Freeze-drying was performed using an Alpha 1-2 LD plus (Christ, Osterode, Germany) to yield the final isolate powder.

### 2.3 Proximate Composition

Proximate composition analyses of chickpea flour and CPI were performed according to AOAC Official Methods^21^ 925.10 (moisture), 923.03 (ash), 920.85 (lipid), and 920.87 (crude protein using % N × 6.25 for chickpea). Chickpea flour and obtained protein isolates were stored at 4 °C until further analyses.

### 2.4 Percent Protein Solubility

Solubility of CPI was determined using the method described by Morr et al.^22^ In brief, 0.2 g of protein isolate was dissolved in 19.8 mL of deionized water using a T25 Ultra-Turrax homogenizer (IKA-Werke GmbH & Co. KG, Staufen, Germany). Afterward, pH was adjusted to 3.0, 5.0, 7.0, and 9.0 using 0.1 M NaOH or 0.1 N HCl in order to observe the effect of pH on CPI solubility. Solutions were then centrifuged at 12,000*g* for 10 min at room temperature. The percent protein solubility was then determined by measuring the nitrogen content using a distillation Unit K-350 (Büchi, Flawil, Switzerland). Protein solubility was calculated by dividing the nitrogen content of the supernatant by the total nitrogen in the protein isolate.

### 2.5 Net Surface Charge (Zeta Potential ζ)

Surface charges of the samples were determined using the method applied by Karaca et al.^20^ In brief, CPI was dissolved in deionized water at 0.05% (w/w), and pH was adjusted using 0.1 N HCl and NaOH. Net surface charge was measured using a Zetasizer (Nano-ZS90, Malvern). Equation 1 was used to calculate net surface charge.
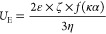
1where ε is the permittivity, *f*(κα) is a function related to the ratio of particle radius (α) and the Debye length (κ), and η is the dispersion viscosity. For this study, the Smoluchowski approximation *f*(κα) equaled 1.5.

### 2.6 Emulsifying Activity and Stability Indices

Emulsifying activity (EAI) and stability (ESI) of CPI was determined using modified version of the method of Pearce and Kinsella.^23^ In brief, 20 mL solutions were prepared dissolving CPI in deionized water (100 mg/L), and by using either 0.1 N HCl or NaOH, pH was adjusted to 7.0. Afterward, solutions were stirred at room temperature for 30 min to completely dissolve protein in water, and 6.5 mL sunflower oil was added. The mixtures were then homogenized using a T25 Ultra-Turrax homogenizer at 4500 rpm for 5 min. Afterward, 200 μL of the emulsions were dispersed in 25 mL SDS solution (10 mg/mL). Emulsion capacity was determined by measuring turbidity at 500 nm using Synergy HT (BioTek Instruments Inc., Vermont, U.S.) and calculation was performed using eq 2
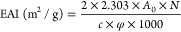
2

Emulsion stability was determined by re-measuring turbidity after 10, 30, and 60 min. ESI was calculated depending on time using eq 3
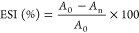
3where *A*_0_ is the initial absorbance value, *N* is the dilution factor (×150), *c* is the weight of protein per volume (g/mL), φ is the oil fraction, *A*_n_ is the absorbance after specific incubation time, and *t* is the time interval, which were 10, 30, and 60 min for ESI.

### 2.7 Creaming Stability Index

Creaming stability of CPI was investigated in two different experimental sets. In the first set of experiments, the effect of pH on creaming stability of CPI was investigated. To determine the creaming stability, the method used by Karaca et al.^20^ was modified. Briefly, 20 mL of mixture was prepared by dispersing CPI in deionized water (1000 mg/L). Afterward, pH was adjusted to 3.0, 5.0, 7.0, and 9.0 using 0.1 M NaOH or 0.1 N HCl. Then, 6.5 mL of sunflower oil was added to the mixture and re-homogenized using a T25 Ultra-Turrax at 9000 rpm for 8 min. Emulsions were then transferred into 10 mL measuring cylinders and held at room temperature (20–23 °C) for 60 min. Creaming stability of each sample was calculated depending on phase separation and calculated by using eq 4
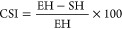
4where EH is the total emulsion height and SH is the serum height.

In the second set, designed experimental mixtures were investigated. In brief, emulsion samples were prepared and pH was adjusted to 7.0 using 0.1 M NaOH. Afterward, the same procedures were applied to determine the creaming stability of the experimental design.

### 2.8 Thermal Analysis

Thermal behavior of CPI was determined by differential scanning calorimetry (DSC). Analysis was performed using a DSC Q10 (TA Instruments, New Castle, DE, USA). Within the thermal scan range of 20–300 °C at a rate of 10 °C min^–1^, the atmosphere used was nitrogen, with a 100 mL/min flow. Calibration was carried out with indium and zinc. An empty pan was used as reference.

### 2.9 Experimental Design

The ranges of components studied were determined based on previous experiments. Before preparing emulsions for the spray-drying process, the effects of CPI and oil concentration on creaming stability were investigated. For this purpose, a central composite design was generated based on response surface methodology (RSM) with Minitab (Minitab, LLC, ver. 19.2020.10, Pennsylvania, USA).

### 2.10 Emulsion Preparation

The effect of CPI, MD, and cumin seed essential oil concentration on the stability of emulsions were investigated using a three-level extreme vertices mixture design and 15 experimental settings were generated with Box–Behnken design with 25–35% for MD, 1–3% for CPI, and 10–20% for cumin seed essential oil concentrations were set. All solutions were prepared by dissolving CPI in deionized water to obtain a final 50 g/L concentration. Each sample was prepared in 150 mL glass containers with 100 g of final weight, and by using 0.1 M NaOH, pH was adjusted to 7.0. All samples were prepared on employing a T25 Ultra-Turrax homogenizer at 9000 rpm for 8 min. Experiments were carried out in triplicate and reported with mean value ± standard deviation. Experimental design, data analysis, and contour plots were carried out using Minitab software (Minitab, LLC, ver. 19.2020.10, Pennsylvania, USA).

### 2.11 Spray Drying

Based on the obtained results of the creaming stability tests, spray-drying experiments were performed to investigate the potential of CPI and MD matrices for encapsulation of cumin seed essential oil. The microencapsulation process was performed using a mini spray drier B-290 (Büchi, Flawil, Switzerland). The inlet air temperature of the spray dryer was adjusted to 135 ± 5 °C, the outlet temperature was kept at 78 ± 3 °C, with an air pressure of 6 bar. To prevent overheating of the samples, the emulsion flow rate and the air flow rate were adjusted depending on the viscosity of samples and ambient temperature. Spray-dried cumin seed essential oil microcapsules were stored at 4 °C in airtight containers until further analyses.

### 2.12 Moisture Content and Water Activity

Moisture content of the microcapsules was determined using an infrared moisture analyzer XM50 (Precisa Gravimetrics AG, Dietlikon, Switzerland) and the water activity (*a*_w_) of the samples was determined using Protimeter (Protimeter, Amphenol Thermometrics, St. Mary’s, USA).

### 2.13 Surface and Total Oil Content

Total oil content of the microcapsules was determined using the method defined by Klinkesorn et al.^24^ with some modifications. In brief, 2 g of microcapsules were diluted with 8 g of deionized water and mixed at 3000 rpm for 2 min. The mixture was then dispersed in 40 mL hexane/2-propanol (3:1 v/v) solution, mixed at 3000 rpm for 5 min, and to obtain phase separation the final solution was centrifuged at 6000*g* for 5 min at room temperature (20–23 °C). The oil-containing upper phase was collected and procedure was re-applied to separate the remaining oil in the solution. Afterward, the collected upper phase was filtered through anhydrous Na_2_SO_4_ to separate water from the solution and then the solvent was allowed to evaporate overnight in a fume hood. Total oil content was measured gravimetrically, after heating the beaker at 105 °C for 30 min to suspend any solvent residue. Oil retention (%) was calculated using eq 5
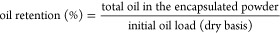
5

Surface oil of content of the microcapsules was determined using the method previously defined by Liu et al.^25^ First, 2 g of microcapsules were dispersed in 30 mL of hexane and then filtered. The same solvent removal procedure was applied for the filtered medium to determine the surface oil content gravimetrically. Cumin seed essential oil EE was calculated using eq 6
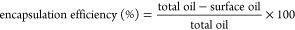
6

### 2.14 Volatile Composition

To determine the major volatile components of the cumin seed essential oil in the microcapsules, the method used for total oil content determination was modified. In brief, approximately 200 mg of microcapsules was mixed with 800 mL of deionized water and vortexed using Vortex 4 basic (IKA-Werke GmbH & Co. KG, Staufen, Germany) for 5 min at 3000 rpm and then mixed at 3000 rpm for 5 min. The final mixture was centrifuged at 6000*g* for 5 min at room temperature (20–23 °C). Then, the organic layer was injected into the tubes for analysis. To specify the major volatile compounds present in both cumin seed essential oil and in microencapsulated samples, a GC–MS QP2020 (Shimadzu Europa GmbH, Duisdurg, Germany) was used employing a previously reported procedure.^26^ The capillary column used for the analysis was GsBP-5MS with dimensions as 30 m × 0.25 mm ID, 0.25 μm, and comprised 5% diphenyl and 95% dimethylpolysiloxane (nonpolar column) with a temperature range of −60 to 350 °C. Helium (He) was used as a carrier gas with the flow rate of 1 mL/min. Approximately 0.2 μL of the cumin seed essential oil samples were injected with a split ratio of 50:1 to avoid overloading of the column. The oven temperature was programmed as 60 °C hold for 2 min initially, then 60 to 260 °C at the rate of 5 °C/min with a total run time of 42 min. The injector was kept at 220 °C and the MS source and inlet line temperature was kept at 280 °C. The mass range was kept from 15 to 350 amu.

### 2.15 Statistical Analyses

Three replicates were measured on duplicate batches of protein isolates. All experiments were reported as the mean ± standard deviation. To measure statistical differences in emulsifying characteristics of CPI and creaming stability as a function of protein and oil concentrations, a two-way analysis of variance (ANOVA) with a Tukey test was used. All statistical analyses were performed with Minitab version 19 software (Minitab, LLC, ver. 19.2020.10, Pennsylvania, USA).

## 3 Results and Discussion

### 3.1 Protein Isolation and Characterization

#### 3.1.1 Proximate Composition of Chickpea Flour and CPI

Proximate compositions of the raw material and obtained isolate are presented in Table 1. The protein content of chickpea flour was comparable with the values previously reported in the literature.^27^ On the other hand, compared to previous studies the protein content of the CPI was quite higher.^20^ Because relatively higher composition of lipid limits the higher protein purity extraction, defatting the sample prior to protein extraction is required to improve protein purity because protein–lipid interactions are significantly reduced.^20,28^

**Table 1:** Proximate Composition of Chickpea Flour and CPI

	protein (%)	moisture (%)	fat (%)	ash (%)	carbohydrate (%)^a^
flour	20.9 ± 0.3^b^	10.6 ± 0.0^a^	5.4 ± 0.0^a^	2.4 ± 0.0a	60.7
CPI	90.9 ± 0.6^a^	3.4 ± 0.0^b^	0.4 ± 0.0^b^	2.0 ± 0.0^b^	3.4

a Calculated by percent differential from 100%. Means in each column followed by different letters were significantly different (*p* < 0.05).

#### 3.1.2 Physicochemical Properties of CPI

The differentiating solubility at various pH values is considered to be a beneficial parameter for the performance of isolates in various food applications. Increased solubility in pH below and above the pI can be associated with decreased protein–protein interaction due to the charged nature of proteins outside of their pI.^29^ Solubility and surface charge of CPI depending on pH change is presented in Table 2. CPI showed the lowest solubility at pH = 5.0 where pI is closest and highest (>80%) at pH = 9.0. Compared to previous studies, quite same CPI solubility was obtained.^30^ Can Karaca et al. reported that the lowest solubility for CPI was at pH = 5.5 (∼4.2%) and the highest at pH = 8.0 and 9.7.^31^ Accordingly, Boye et al. also reported high (80–90%) solubility values for chickpea and lentil protein isolates at pH = 1.0–3.0 and at pH = 7.0–10.0.^8^ Differences observed in protein solubility are attributed to the variations in the surface amino acid composition of proteins. In addition, the solubility profile of the proteins offers some perspective of the extent of denaturation or irreversible aggregation and precipitation which might have occurred during the isolation process.^29^ Emulsifying properties are generally classified as emulsion activity, which represents the ability of the proteins to lead formation and stabilization of the recently formed emulsion, and emulsion stability, which represents the ability of the proteins to implement strength against phase separation on the emulsion.^32^ EAI, ESI, and creaming stability index (CSI) of CPI are presented in Table 2. While the highest EAI (61.78 m^2^/g) and the lowest CSI (9.83%) were observed at pH = 9.0, the highest ESI (123.78 min) was observed at pH = 7.0. Regarding the solubility of CPI, in Table 2, increased solubility of CPI in the pH range of 5.0–9.0 has a positive effect on CSI. Low CSI values are distinctive of low serum separation, which indicates higher emulsion stability.

**Table 2:** Net Surface Charge, Solubility, and Emulsifying Properties of CPI

pH	percent CPI solubility	surface charge (mV)	EAI (m^2^/g)	ESI (min)	CSI (%)
3.0	35.7 ± 3.1^c^	29.4 ± 1.4^a^	7.21 ± 0.18^d^	42.41 ± 4.27^c^	30.50 ± 0.55^a^
5.0	25.7 ± 0.0^d^	–15.4 ± 2.3^b^	11.83 ± 0.11^c^	24.75 ± 1.27^d^	27.33 ± 0.52^b^
7.0	44.7 ± 3.2^b^	–31.9 ± 1.9^c^	31.20 ± 0.31^b^	125.29 ± 4.47^a^	22.42 ± 0.49^c^
9.0	94.4 ± 2.1^a^	–33.9 ± 1.1^c^	61.78 ± 0.62^a^	94.74 ± 6.16^b^	9.83 ± 0.75^d^

Slightly lower emulsion stability was observed at pH = 3.0 (in terms of cream separation of 30.50%) compared to pH = 5.0 (27.33% cream separation), although the protein solubility was higher at pH = 3.0. The emulsions exhibited more extensive droplet flocculation, more viscous, and more structured, but less creaming stability at low ionic strength compared to higher ionic strength^33^ which associates differences in both pH on EAI, ESI, and CSI. Although significantly higher protein solubility was observed at pH = 3.0, higher emulsifying activity and lower creaming stability were observed at pH = 5.0. Emulsions prepared at pH = 9.0 showed lower reduction on EAI over time compared to pH = 7.0 (Table 2). As the pH drift apart from the pI, while ζ-potential and repulsions increasing, flocculation decreases. Electrical charges on lipid droplets may increase due to the ionization, absorption, or frictional electricity as a result of the large shearing forces caused by the emulsification process.^34^ In addition, overall emulsion stability was also investigated with increasing CPI concentration, 0.10, 0.25, 0.50, and 1 g of CPI was diluted with 100 mL deionized water, at pH = 7.0. The highest isolate containing solution had the highest EAI change over time. This might be a result of emulsifying properties of CPI being reduced consequently aggregation or precipitation of protein and affiliated with loss of colloidal stabilizing characteristics, due to high concentrations of electrolytes.^35^

Emulsifying properties of proteins are complex and depends on many parameters such as total molar mass, hydrophobicity/solubility, conformation stability, and charge and physicochemical factors (e.g., tertiary or quaternary structures), temperature, protein concentration, amino acid profile, and pH, and accordingly the ionic strength.^32^ EAI most commonly depends on the quaternary conformation of the protein and its flexibility. Besides, ESI depends on tertiary conformation of the protein and its flexibility. For soy protein isolate, emulsification capacity of the isolate was reported to decrease as the purity of the protein isolate increased. Principal component analysis was reported to show that EAI was positively influenced by thermal properties (e.g., *T*_d_ and Δ*H*_d_), which are also affected by the structural properties of the protein.^36^

**Figure 1:**
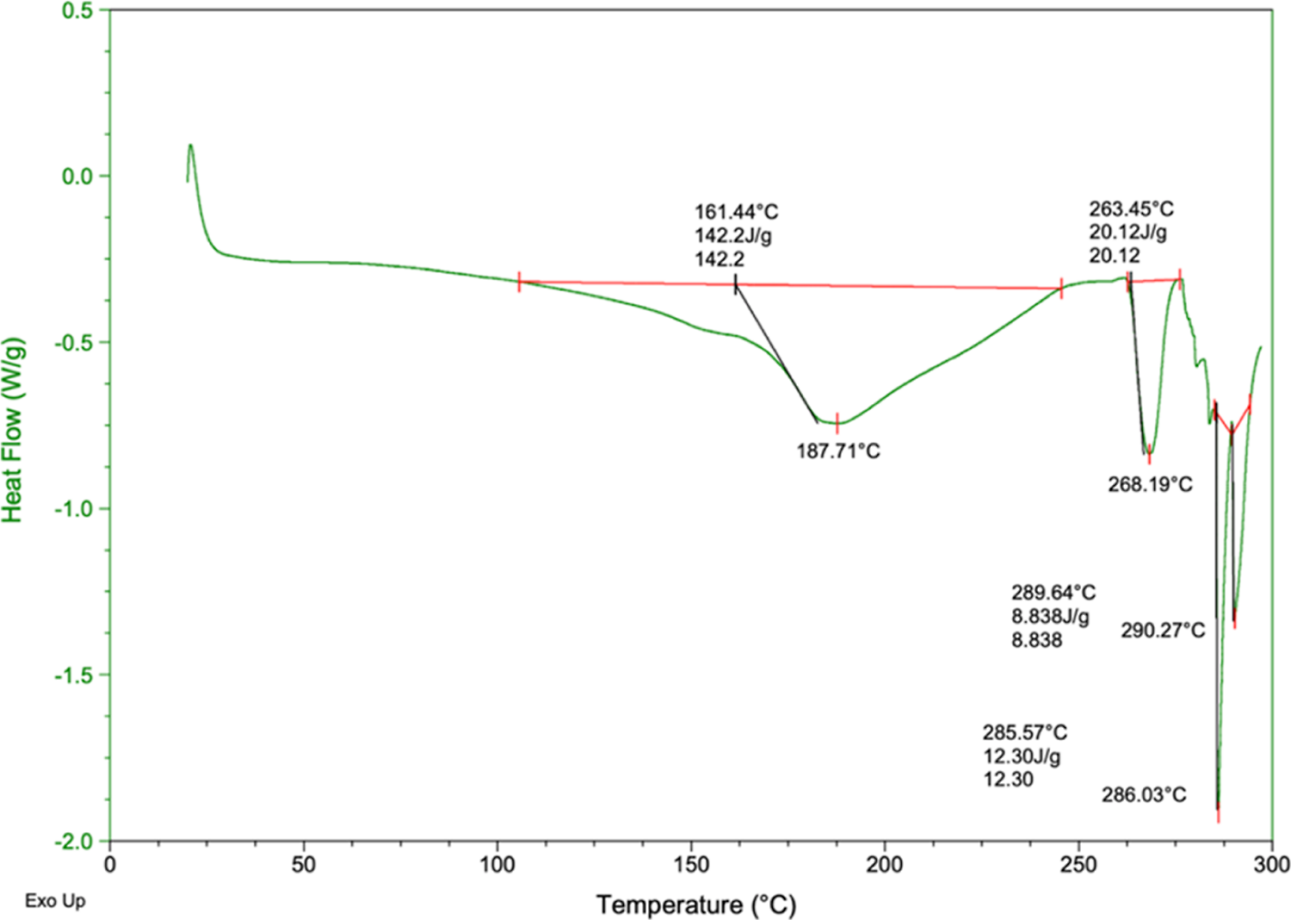
DSC thermogram of CPI in the temperature range of 0–300 °C (3.43% moisture content).

Figure 1 shows the thermal behavior of CPI as a function of temperature. CPI (90.88% protein) showed the greatest enthalpy changes (Δ*H*_d_) (142.2 J/g) during the first endothermic event at a denaturation temperature (*T*_d_) of 187.71 °C, followed by 268.19 °C. The enthalpy changes reflected the extent of ordered structure of the globulin as the transition from native to denatured state took place.^37^ High *T*_d_ indicates heat-resistant protein fractions,^36^ according to previous studies; prolamine films occur at 228, 250, and 270 °C for rice, wheat, and soybeans, respectively.^38^ The moisture content of the isolate, or the solution affects *T*_d_. For dissolved proteins in solution, *T*_d_ is usually below 100 °C. Depending on the amino acid composition of the protein, as much as the moisture content decreases, required temperature and the enthalpy increases. Furthermore, thermal characteristics of globular proteins are additionally requisite, due to their heat-dependent aggregation and gelation behaviors. A higher *T*_d_ is more commonly related to higher thermal stability for a globular protein. Moreover, disruption of hydrogen bonds maintaining tertiary and quaternary structures of proteins, especially tertiary ones, is also demonstrated.^39,40^

#### 3.1.3 Optimization and Method Validation of CPI-Stabilized Emulsions

Optimum protein concentration for CPI-stabilized emulsions was determined using an experimental design (Table 3). For CPI-stabilized emulsions, a predictive model for estimating its CSI correlated with the following factors: CPI, CPI*CPI, oil, and oil*oil. The obtained model was able to predict 82.21% of data variability. Around these parameters, it was observed that oil*oil had the maximum effect on CSI, followed by oil itself. Meanwhile, no significant effect of the CPI*oil coefficient was observed.

**Table 3:** RSM Design of CPI-Stabilized Emulsions

run order	CPI (%)	oil (%)	CSI (%)
1	1.25	12.50	20.50 ± 1.05^de^
2	1.25	23.11	90.00 ± 0.63^j^
3	0.50	20.00	33.50 ± 1.05^h^
4	1.25	12.50	21.50 ± 1.05^e^
5	1.25	12.50	19.80 ± 0.75^d^
6	0.19	12.50	24.00 ± 0.89^f^
7	0.50	5.00	5.20 ± 0.75^b^
8	1.25	12.50	20.50 ± 0.55^de^
9	2.00	5.00	17.50 ± 0.84^c^
10	1.25	1.89	0.00 ± 0.00^a^
11	2.31	12.50	20.00 ± 0.63^de^
12	1.25	12.50	25.70 ± 0.65^g^
13	2.00	20.00	36.70 ± 0.52^i^

Lower CSI exhibits lower serum separation, indicating higher stability of the emulsion. Experimental results showed that the lowest CSI (0–10%) was observed where the CPI concentration was 0–0.5% and the oil concentration was 0–12%. According to Stokes’ law, emulsions with higher stability and lower tendency to phase separation, can be obtained with smaller droplet sizes, lower density contrast between phases, and higher phase viscosities. The creaming rate of the emulsion can be minimized by increasing the protein concentration which decreases the density difference between the oil and water phases of the emulsion.^31^ As the oil concentration of the emulsion increases, a concomitant increase in oil droplet packing occurs. Increase in the mobility of oil leads to increase in emulsion coalescence and separation due to decrease in viscosity of the continuous phase.^41^ In addition, when the CPI concentration increased from 0.5%, CSI of the prepared emulsions increased from 0–10% to 10–20% at constant oil concentrations (0–12%) because proteins at the interface generally unfold, which is affected by amino acid composition, and distribution of the nonpolar residues in a protein chain, due to the lower protein concentration.^34^ Because all emulsions were prepared at pH = 7.0, the main affecting force for the emulsion stability is steric, rather than electrostatic.^42^ Furthermore, at pH values above the pI, surfaces of the globules are charged negatively due to negatively charged carboxyl groups of proteins.^43^ Additionally, as the interactions between two droplets increase, globules become more resistant to desorption. RSM was used to achieve minimum CSI (4.16%) with initial 0.19% CPI, and 6.83% oil concentrations on the prepared emulsion. Designed experiment resulted in 0.00% CSI with no phase separation after 1 h of incubation.

### 3.2 Microencapsulation of Cumin Seed Oil Using CPI and MD

#### 3.2.1 Microcapsule Characteristics

Table 4 shows the °Brix value of the emulsions, before the spray-drying application, and the moisture content and water activity of the collected microcapsules. The moisture content of the microcapsules ranged between 3.17 and 5.24% (*p* < 0.05), and their water activity varied from 0.16 to 0.25 (Table 4). Meanwhile *a*_w_ of all samples fit the industrial *a*_w_ specification, which is < 0.3, the moisture content of some dried samples exceeds the common specifications, 3–4%.^24^ A relatively high moisture content of the spray-dried powders is attributed to the higher bulking weight as a result of the presence of higher amounts of water on the emulsion.^44^ °Brix, moisture content, and *a*_w_ of run order 5, 6, and 8 were found to be quite similar which had the same concentration of CPI 2%, cumin seed essential oil 15%, and MD 30%.

**Table 4:** Brix of Feed Emulsions and Moisture Content, Water Activity, Surface Oil, Oil Retention, and EE of Spray-Dried Cumin Seed Essential Oil Samples^a^

run order	CPI (%)	cumin oil (%)	MD (%)	°Brix	moisture content (%)	water activity	surface oil (%)	oil retention (%)	EE (%)
1	3	15	25	27.67 ± 0.58^hi^	3.52 ± 0.04^h^	0.19 ± 0.01^fg^	1.4 ± 0.04^i^	91.5 ± 0.86^bc^	98.4 ± 0.05^a^
2	1	15	35	31.67 ± 0.58^d^	3.85 ± 0.04^g^	0.24 ± 0.01^ab^	1.4 ± 0.04^i^	91.7 ± 1.24^abc^	98.4 ± 0.06^a^
3	2	10	35	32.83 ± 0.76^cd^	3.54 ± 0.03^h^	0.21 ± 0.01^cdef^	2.4 ± 0.00^h^	94.6 ± 1.06^abc^	97.4 ± 0.03^bc^
4	3	15	35	36.00 ± 0.00^b^	4.07 ± 0.03^f^	0.25 ± 0.01^a^	2.3 ± 0.04^h^	93.9 ± 0.75^abc^	97.6 ± 0.04^b^
5	2	15	30	32.17 ± 0.29^cde^	5.24 ± 0.02^a^	0.21 ± 0.01^cde^	2.8 ± 0.01^g^	94.1 ± 0.96^abc^	97.0 ± 0.02^cd^
6	2	15	30	32.67 ± 0.58^cd^	4.85 ± 0.03^b^	0.21 ± 0.01^cde^	2.9 ± 0.00^g^	95.1 ± 1.52^ab^	96.9 ± 0.05^d^
7	1	20	30	30.67 ± 0.58^ef^	4.51 ± 0.01^c^	0.22 ± 0.01^bc^	5.4 ± 0.09^d^	91.7 ± 2.50^abc^	94.1 ± 0.07^g^
8	2	15	30	32.17 ± 0.29^cde^	4.82 ± 0.02^b^	0.21 ± 0.01^cde^	2.9 ± 0.29^g^	94.5 ± 1.10^abc^	96.9 ± 0.33^cd^
9	3	20	30	32.50 ± 0.87^cde^	5.16 ± 0.05^a^	0.23 ± 0.01^abc^	4.8 ± 0.02^e^	96.4 ± 2.57^a^	95.0 ± 0.11^f^
10	2	10	25	26.33 ± 0.58^i^	3.38 ± 0.03^i^	0.20 ± 0.01^efg^	6.9 ± 0.04^b^	90.2 ± 1.96^c^	92.4 ± 0.13^i^
11	2	20	35	39.67 ± 0.58^a^	3.56 ± 0.05^h^	0.16 ± 0.01^h^	3.5 ± 0.10^f^	90.4 ± 1.03^cd^	96.2 ± 0.13^e^
12	3	10	30	33.67 ± 0.58^c^	4.36 ± 0.04^d^	0.18 ± 0.01^g^	7.9 ± 0.06^a^	86.6 ± 1.17^d^	90.9 ± 0.10^k^
13	1	15	25	28.67 ± 0.58^gh^	4.20 ± 0.01^e^	0.20 ± 0.01^defg^	7.8 ± 0.18^a^	93.8 ± 2.06^abc^	91.7 ± 0.30^j^
14	1	10	30	32.17 ± 0.29^cde^	3.17 ± 0.06^j^	0.22 ± 0.01^cde^	6.1 ± 0.17^c^	92.8 ± 1.47^abc^	93.4 ± 0.23^h^
15	2	20	25	29.67 ± 1.15^fg^	5.21 ± 0.03^a^	0.22 ± 0.01^cd^	5.6 ± 0.23^d^	92.6 ± 1.39^abc^	93.9 ± 0.28^gh^

a Means in each row followed by different letters were significantly different (*p* < 0.05).

The highest EE and lowest surface oil were observed at initial composition of oil concentration of 15%, following CPI concentration of 3 and 1%, and MD concentration of 25 and 35% (RO 1 and 2); with values of 98.4% EE and 1.4% surface oil. On the other hand, the highest oil retention (96.4%), was observed at initial composition of oil load of 20%, CPI concentration of 3%, and MD concentration of 30% (RO 9).

For CPI-coated microcapsules, a predictive model for estimating oil retention and EE supported the inclusion of the following factors: the essential oil concentration, the CPI concentration, and the MD concentration. The obtained model was able to predict 71.13% for oil retention and 83.68% for EE of data variability. Comparing affecting parameters on oil retention, it was observed that CPI*cumin oil had the maximum effect on oil retention, followed by cumin oil*cumin oil, and CPI and MD had the minimum effect on the oil retention. Besides, when significant parameters were compared, it was observed that MD (*p* < 0.1) itself has the maximum effect on EE, followed by CPI*MD and cumin oil*cumin oil. Additionally, MD had the maximum effect on lower surface oil, followed by CPI*MD. As the interaction between hydrophobic side of the CPI and essential oil increase, CPI provides better lipid bounding on the system, while MD itself and its interaction with CPI provides better emulsion forming and thus encapsulation yield because they hold essential oil in the complex matrix.

At different MD values, it was observed that for a constant CPI concentration, both oil retention and EE first increased with an increasing cumin seed essential oil concentration and then decreased. As the oil concentration of the emulsion increases with constant wall material concentration, oil droplet packing occurs with increasing ratios^45^ and emulsion reaches maximum oil holding capacity which leads to decrease in both oil retention and EE. Because complex matrices produced with protein and MD are uniform after spray-drying operation, it provides a high oil retention rate and produces a powder with low hygroscopicity.^46^ Furthermore, for constant cumin seed essential oil concentration, similar phenomena were observed in oil retention, which may be caused by the lack of MD concentration on the pre-emulsion in proportion to theCPI concentration.

#### 3.2.2 Optimization and Method Validation

Depending on experimental results, a new experimental set with 14.75% CPI, 35% MD, and 14.75% cumin seed essential oil concentrations was performed for method validation, which predicted the following: oil retention of 94.31% and EE of 98.42%. Designed experiment led to microcapsule sample with a moisture content of 3.46 ± 0.06% and 0.17 ± 0.01*a*_w_. Collected sample had 1.30 ± 0.19% surface oil, 92.89 ± 1.26% oil retention, and 98.60 ± 0.23% EE. Compared to the predicted values, slightly lower oil retention was obtained. On the other hand, obtained EE was higher than the predicted value. Compared to Box–Behnken design, obtained EE was among the highest values. In optimized set, the lowest surface oil content was achieved.

#### 3.2.3 Volatile Composition of Free and Encapsulated Cumin Seed Oil

Volatile composition of microencapsulated cumin seed essential oil determined by GC–MS analysis is presented in Table 5. Among the chemical compounds observed, highest 14 compounds were selected as key components for further comparison. Considering the results of optimized run and initial Box–Behnken design runs, it was observed that optimized design provided better protection against the changes in volatile composition during the spray-drying operation (Table 5). Comparing the major compounds present in cumin seed essential oil, terpineol (81.66%) showed the maximum residual in optimized microcapsules. Within the Box–Behnken experimental design samples, optimized formulation resulted in higher α-pinene (59.09%), β-pinene (53.88%), cymene (55.88%), terpinene (63.72%), and cumin aldehyde (68.10%) in spray-dried cumin seed essential oil microcapsules.

**Table 5:** Volatile Composition of Microencapsulated Cumin Seed Essential Oil Samples

	R1	RO2	RO3	RO4	RO5	RO6	RO7	RO8	RO9	RO10	RO11	RO12	RO13	RO14	RO15	optimized
*o*-xylene	62.74	63.15	85.52	58.78	45.75	47.89	43.38	47.55	33.89	57.51	45.34	57.62	57.12	74.08	26.42	65.54
*m*-xylene	60.69	62.58	86.72	58.71	46.16	48.26	43.59	47.60	34.40	58.74	46.01	57.99	56.55	74.67	27.08	66.80
α-pinene	15.30	55.02	54.40	43.85	22.43	29.39	23.43	26.18	23.32	35.57	23.88	41.93	24.47	51.45	33.17	59.09
sabinene	16.77	67.21	61.41	49.57	21.38	31.81	27.41	29.38	26.79	40.47	25.16	43.73	41.87	53.98	36.02	63.95
β-pinene	16.46	40.43	50.37	41.29	22.99	29.71	24.17	26.37	23.28	35.71	24.37	40.27	25.30	48.97	29.66	53.88
β-myrcene	21.84	67.42	65.09	51.74	23.41	31.15	29.19	31.41	28.57	43.00	25.13	42.60	47.55	54.62	35.42	63.18
phellandrene	27.56	78.90	84.04	66.72	24.90	32.56	38.56	40.92	36.21	55.35	26.36	44.40	54.12	56.18	36.18	65.10
*p*-cymene	18.78	39.61	49.36	38.83	24.27	31.86	24.64	26.45	22.56	35.01	25.51	41.02	30.12	50.59	30.39	55.88
terpinene	20.84	57.68	59.11	46.63	23.12	30.79	26.74	27.49	25.23	38.61	24.77	43.07	40.12	53.17	34.51	63.72
Δ-carene	21.58	90.88	65.85	53.77	23.56	31.63	30.77	31.98	28.75	45.41	24.78	42.26	46.17	52.62	34.01	64.12
γ-terpinene	23.33	49.79	60.92	48.45	24.47	31.52	29.62	31.12	27.39	42.25	26.08	41.93	42.28	52.46	32.36	58.48
α-terpineol	17.15	51.67	56.86	51.11	32.59	46.96	37.58	37.76	33.60	47.25	35.56	53.39	50.19	60.79	43.29	81.66
cumin aldehyde	19.55	37.37	51.28	45.75	32.93	45.31	36.20	36.13	31.31	44.16	34.72	49.32	45.12	55.02	36.32	68.10
carvone	31.21	46.98	53.01	57.11	45.45	66.48	50.24	49.52	45.18	56.26	49.63	62.61	57.25	66.21	50.63	97.65

## 4 Conclusions

Overall, the emulsion formation and stabilization properties of CPI were affected by the protein concentration, pH of the medium, net surface charge, and solubility. When CPI was used in combination with MD for stabilizing cumin seed essential oil emulsions, at constant MD concentrations, CPI had positive effects on emulsion formation and stability. With increasing cumin seed essential oil and CPI concentrations, higher oil retention and EE were achieved. However, at a constant MD concentration, when the emulsion reached the maximum oil holding capacity, stability of the emulsion decreased due the lack of the MD concentration in the medium and increase in oil droplet packing. Increasing the MD concentration in emulsion depending on CPI and cumin seed essential oil concentrations provided better stability. Moreover, using MD in combination with CPI in emulsion as a wall material provided not only higher EE but also a better protecting layer around the cumin seed essential oil globules against degradation when particles were exposed to hot airflow during the spray-drying operation. Furthermore, even after the relatively high-temperature spray-drying application, cumin seed essential oil better maintained its volatile composition. Optimized emulsion formulation resulted in improved EE and minimal changes in volatile composition of cumin seed essential oil.

## Supporting Information

Contour plots, GC–MS profile, and predictive models (PDF)
