# Supplementary material for: Encapsulation of Cumin (Cuminum cyminum L.) Seed Essential Oil in the Chickpea Protein–Maltodextrin Matrix
Source: ACS Omega. 2023 Jan 18;8(4):4156–64. doi: 10.1021/acsomega.2c07184 (PMC9893743; doi:10.1021/acsomega.2c07184)
Supplement: Supplementary file 1 — ao2c07184_si_001.pdf [file ao2c07184_si_001.pdf]

Supporting Information:
Encapsulation of Cumin (*Cuminum cyminum* L.) Seed Essential Oil in Chickpea Protein-Maltodextrin Matrix

Onur Atli, Asli Can Karaca*, Beraat Ozcelik

Department of Food Engineering, Faculty of Chemical and Metallurgical Engineering, Istanbul Technical University, 34469 Istanbul, Turkey, atlio@itu.edu.tr, cankaraca@itu.edu.tr, ozcelik@itu.edu.tr

* **Corresponding Author**
Asli Can Karaca, Ph.D.
Department of Food Engineering, Faculty of Chemical and Metallurgical Engineering, Istanbul Technical University, 34469 Istanbul, Turkey, E-mail: cankaraca@itu.edu.tr

List of figures

Figure S1. Contour plot indicating changes in creaming stability index (%) with chickpea protein isolate and oil concentration.

Figure S2. Contour plots of oil retention (A - C) and encapsulation efficiency (D - F) of cumin seed essential oil based on cumin seed essential oil and chickpea protein isolate concentration at varying maltodextrin concentrations.

Figure S3. GC-MS profile of cumin seed essential oil.

List of tables

Table S1. Predictive model for estimating creaming index for chickpea protein isolate-stabilized emulsions.

Table S2. Predictive model for estimating oil retention and encapsulation efficiency for chickpea protein isolate and microcapsules.

Table S3. Predictive model for volatile composition of microcapsules.

Figure S1

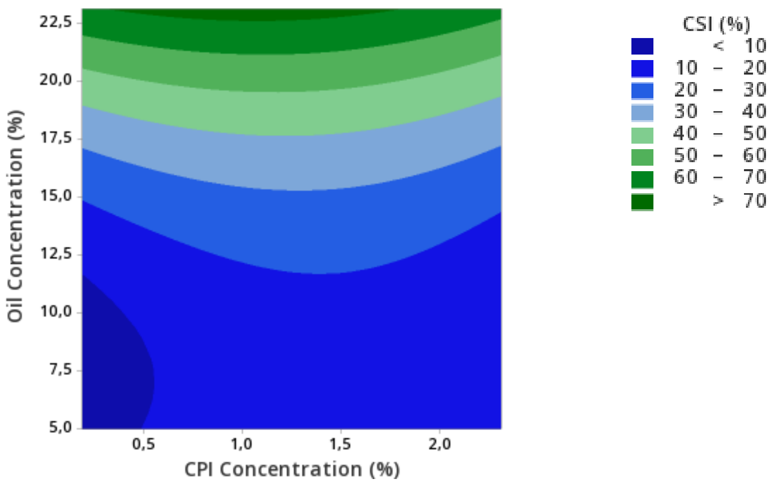
CSI (%)
< 10
10 – 20
20 – 30
30 – 40
40 – 50
50 – 60
60 – 70
> 70
Oil Concentration (%)
22,5
20,0
17,5
15,0
12,5
10,0
7,5
5,0
0,5
1,0
1,5
2,0
CPI Concentration (%)

Figure S2

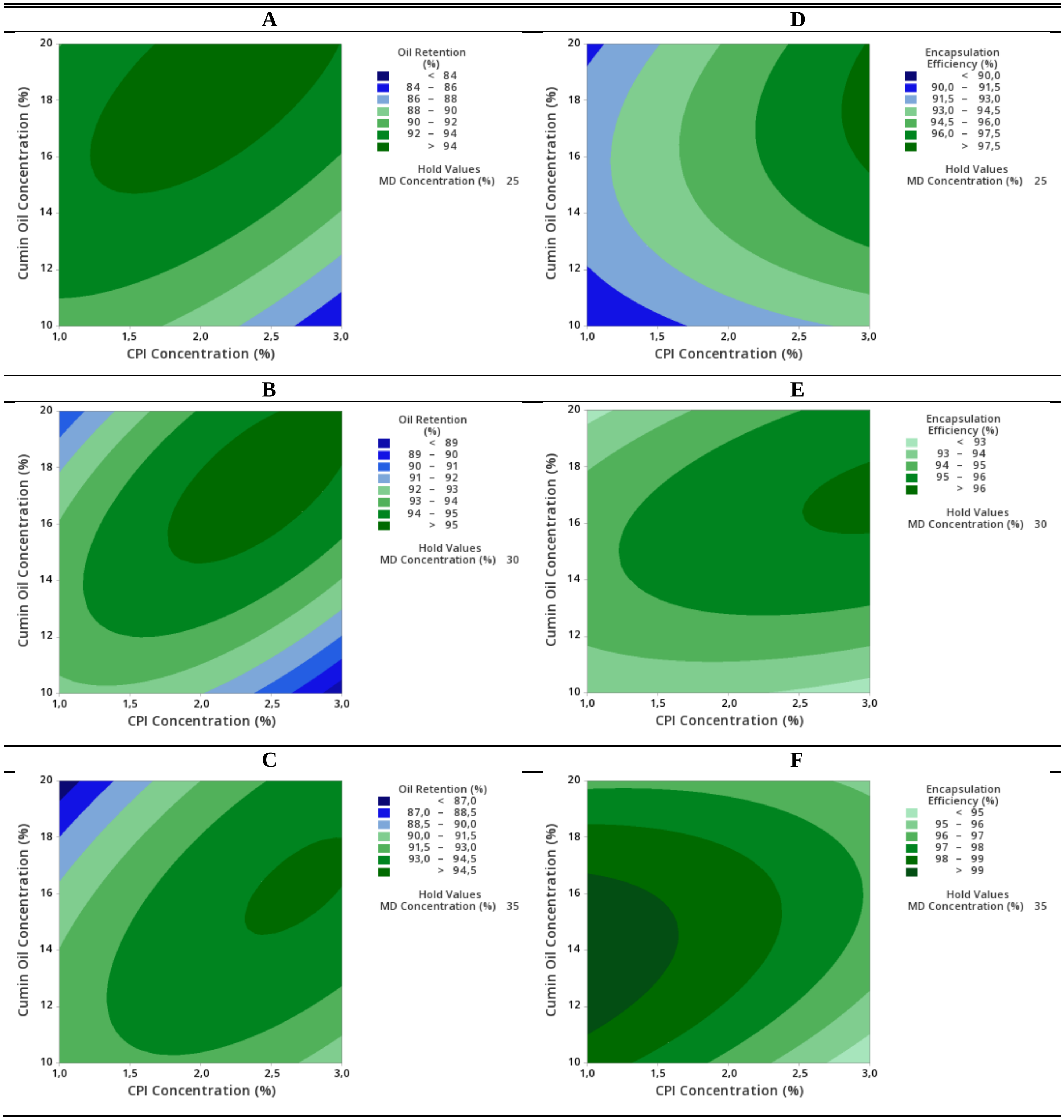
A
Oil Retention (%)
< 84
84 – 86
86 – 88
88 – 90
90 – 92
92 – 94
> 94
Hold Values
MD Concentration (%) 25
Cumin Oil Concentration (%)
CPI Concentration (%)
D
Encapsulation Efficiency (%)
< 90,0
90,0 – 91,5
91,5 – 93,0
93,0 – 94,5
94,5 – 96,0
96,0 – 97,5
> 97,5
Hold Values
MD Concentration (%) 25
Cumin Oil Concentration (%)
CPI Concentration (%)
B
Oil Retention (%)
< 89
89 – 90
90 – 91
91 – 92
92 – 93
93 – 94
94 – 95
> 95
Hold Values
MD Concentration (%) 30
Cumin Oil Concentration (%)
CPI Concentration (%)
E
Encapsulation Efficiency (%)
< 93
93 – 94
94 – 95
95 – 96
> 96
Hold Values
MD Concentration (%) 30
Cumin Oil Concentration (%)
CPI Concentration (%)
C
Oil Retention (%)
< 87,0
87,0 – 88,5
88,5 – 90,0
90,0 – 91,5
91,5 – 93,0
93,0 – 94,5
> 94,5
Hold Values
MD Concentration (%) 35
Cumin Oil Concentration (%)
CPI Concentration (%)
F
Encapsulation Efficiency (%)
< 95
95 – 96
96 – 97
97 – 98
98 – 99
> 99
Hold Values
MD Concentration (%) 35
Cumin Oil Concentration (%)
CPI Concentration (%)

.

Figure S3

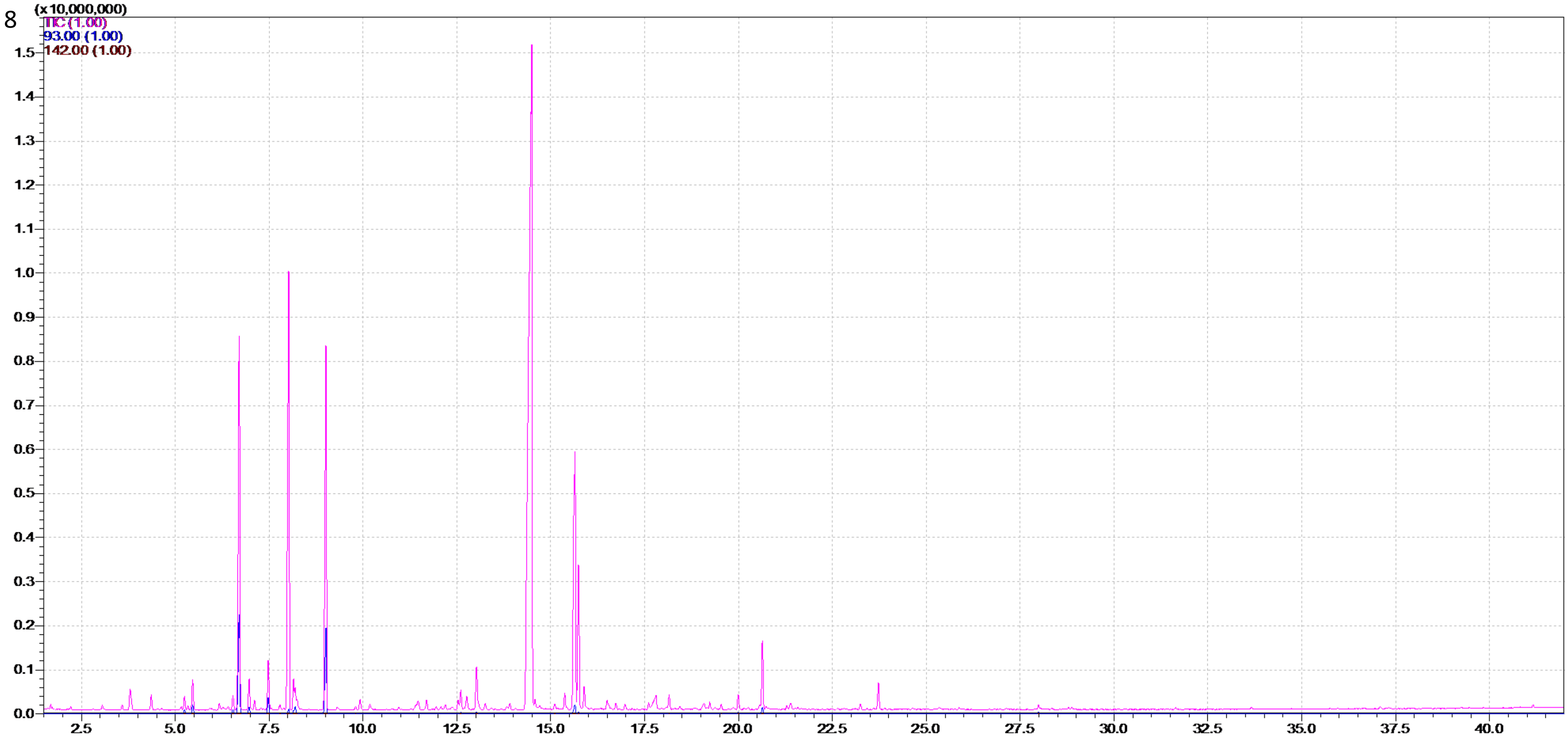
8
(x10,000,000)
TIC (1.00)
93.00 (1.00)
142.00 (1.00)
1.5
1.4
1.3
1.2
1.1
1.0
0.9
0.8
0.7
0.6
0.5
0.4
0.3
0.2
0.1
0.0
2.5
5.0
7.5
10.0
12.5
15.0
17.5
20.0
22.5
25.0
27.5
30.0
32.5
35.0
37.5
40.0

Table S1

independent variables	coefficient	p value	model fit
CPI	6.09	$p<0.5$	$R^2=0.8221$
CPI*CPI	-6.88	$p<0.5$	$F=12.0393$
Oil	12.40	$p<0.5$	$p=0.01$
Oil*Oil	24.20	$p<0.1$	
CPI*Oil	-4.00	NS*	
Constant	16.15		

*NS, not significant ($p>0.5$)

Table S2

Dependent variables	independent variables	coefficient	p value	model fit
Oil retention	CPI	0.138	NS*	R^2=0.7113
	Cumin Oil	1.036	p<0.5	F=2.477
	MD	0.164	NS	p=0.01
	CPI*CPI	-1.55	p<0.01	
	Cumin Oil*Cumin Oil	-2.04	p<0.5	
	MD*MD	-1.10	p<0.5	
	CPI*Cumin Oil	2.35	p<0.5	
	CPI*MD	1.45	p<0.5	
	Cumin Oil*MD	-1.66	p<0.5	
	Constant	95.09	0.000	
Encapsulation efficiency	CPI	0.554	p<0.5	R^2=0.8368
	Cumin Oil	0.648	p<0.5	F=1.630
	MD	1.654	p<0.5	p=0.01
	CPI*CPI	-0.378	NS	
	Cumin Oil*Cumin Oil	-1.930	p<0.1	
	MD*MD	1.237	p<0.5	
	CPI*Cumin Oil	0.830	p<0.5	
	CPI*MD	-1.903	p<0.1	
	Cumin Oil*MD	-0.695	p<0.5	
	Constant	95.663	0.000	

*NS, not significant (p>0.5)

Table S3

independent variables	coefficient	*p* value	model fit	independent variables	coefficient	*p* value	model fit
	Cymene				*A-pinene*		
CPI	-2.97	p<05	R^2=*0.9129*	CPI	-3.75	p<0.5	R^2=*0.8401*
Cumin Oil	-9.11	p<0.005	F=4.7472	Cumin Oil	-9.94	p<0.05	F=8.5921
MD	4.88	p<0.05	p=0.01	MD	8.58	p<0.05	p=0.01
CPI*CPI	1.97	p<0.5		CPI*CPI	3.47	p<0.5	
Cumin Oil* Cumin Oil	5.20	p<0.1		Cumin Oil* Cumin Oil	5.56	p<0.5	
MD*MD	2.34	p<0.5		MD*MD	5.19	p<0.5	
CPI*Cumin Oil	1.87	p<0.5		CPI* Cumin Oil	2.35	NS	
CPI*MD	2.64	p<0.5		CPI*MD	-0.50	NS	
Cumin Oil*MD	-4.81	p<0.1		Cumin Oil*MD	-7.03	p<0.5	
Constant	27.53	0		Constant	26	p<0.005	
	B-pinene				*Sabinene*		
CPI	-2.20	p<0.5	R^2=0.8968	CPI	-6.70	p<0,5	R^2=0.8459
Cumin Oil	-9.23	p<0.005	F=5.5087	Cumin Oil	-10.53	p<0,05	F=9.7594
MD	6.17	p<0.05	p=0.01	MD	8.53	p<0,1	p=0.01
CPI*CPI	1.83	NS		CPI*CPI	6.77	p<0,5	
Cumin Oil* Cumin Oil	5.99	p<0.1		Cumin Oil* Cumin Oil	3.68	NS	
MD*MD	2.68	p<0.5		MD*MD	9.56	p<0,5	
CPI*Cumin Oil	1.95	NS		CPI*Cumin Oil	2.41	NS	
CPI*MD	2.42	p<0.5		CPI*MD	1.87	NS	
Cumin Oil*MD	-4.99	p<0.5		Cumin Oil*MD	-7.95	p<0,5	
Constant	26.36	0		Constant	27.52	0,007	
	Terpinene				*Terpineol*		
CPI	-5.51	p<0.1	R^2=0.9120	CPI	-5.62	p<0.5	R^2=0.8081
Cumin Oil	-10.34	p<0.01	F=6,3890	Cumin Oil	-8.532	p<0.05	F=8.4402
MD	6.49	p<0.05	p=0,01	MD	4.67	p<0.5	p=0.01
CPI*CPI	6.26	p<0.5		CPI*CPI	2.01	NS	
Cumin Oil* Cumin Oil	3.66	p<0.5		Cumin Oil* Cumin Oil	5.22	p<0.5	
MD*MD	8.46	p<0.1		MD*MD	1.41	NS	
CPI* Cumin Oil	2.15	NS		CPI*Cumin Oil	0.86	NS	
CPI*MD	2.60	p<0.5		CPI*MD	8.12	p<0.5	
Cumin Oil*MD	-7.56	p<0.1		Cumin Oil*MD	-4.33	p<0.5	
Constant	27.13	0.002		Constant	39.10	0.001	
	Phellandrene				*Cumin Aldehyde*		
CPI	-6.61	p<0.5	R^2=0.8378	CPI	-3.47	p<0.5	R^2=0.8900
Cumin Oil	-12.83	p<0.05	F=12.5192	Cumin Oil	-7.65	p<0.01	F=5.0645
MD	10.35	p<0.1	p=0.01	MD	3.00	p<0.5	p=0.01
CPI*CPI	8.69	p<0.5		CPI*CPI	0.08	NS	
Cumin Oil* Cumin Oil	2.35	NS		Cumin Oil* Cumin Oil	4.76	p<0.5	
MD*MD	15.34	p<0.1		MD*MD	-1.26	NS	
CPI*Cumin Oil	2.36	NS		CPI*Cumin Oil	0.20	NS	
CPI*MD	3.59	NS		CPI*MD	8.49	p<0.05	
Cumin Oil*MD	-9.63	p<0.5		Cumin Oil*MD	-2.18	p<0.5	
Constant	32.79	0.012		Constant	38.12	0	

*NS, not significant (p>0.5)
